# The use of bilateral orthotopic lung transplantation in the management of acute severe drug-induced interstitial lung disease: A case report

**DOI:** 10.1016/j.jhlto.2024.100108

**Published:** 2024-05-10

**Authors:** Derek K. Afflu, Brittany A. Cody, Elizabeth Lendermon, Pablo G. Sanchez

**Affiliations:** a Division of Lung Transplant and Lung Failure, University of Pittsburgh Medical Center, Pittsburgh, Pennsylvania; b Department of Pathology, University of Pittsburgh Medical Center, Pittsburgh, Pennsylvania; c Division of Pulmonary, Allergy and Critical Care Medicine, University of Pittsburgh Medical Center, Pittsburgh, Pennsylvania

**Keywords:** drug-induced interstitial lung disease, extracorporeal membrane oxygenation, lung transplantation, trimethoprim-sulfamethoxazole (Bactrim), pneumonitis

## Abstract

This case report presents a unique and severe manifestation of drug-induced interstitial lung disease (DIILD) in an 18-year-old male with a recent history of coronavirus disease 2019 (COVID-19) infection. The patient, treated with trimethoprim-sulfamethoxazole (Bactrim) for acne, developed a rapid and aggressively progressive respiratory failure and underwent bilateral orthotopic lung transplantation. Radiologic evaluation revealed extensive bilateral bronchiectasis, ground-glass opacities/consolidation, and fibrotic changes, with diffuse vascular thrombosis observed during explant pathology. Sulfonamides, particularly Bactrim, are implicated in DIILD, with male sex and pre-existing lung disease as common risk factors. The patient's simultaneous exposure to COVID-19 and genetic susceptibility to Bactrim allergy (human leukocyte antigen (HLA) B07:02 and HLA C07:02) may have contributed to the rapid progression of pulmonary fibrosis. Despite limited literature on lung transplantation for Bactrim-induced lung fibrosis, this case underscores its consideration as a viable treatment option in refractory cases.

Interstitial lung disease has numerous causes, with pharmacologic agents such as sulfonamides, implicated in drug-induced interstitial lung disease (DIILD).^1^ Despite typically being self-limiting, we report a case of severe progressive pulmonary reaction to Bactrim in a young adult resulting in lung transplantation.

An 18-year-old male presented in March with fever and shortness of breath following a 1-month course of trimethoprim-sulfamethoxazole (Bactrim) for acne. The patient had been diagnosed with coronavirus disease 2019 (COVID-19) in January before initiating the antibiotic in February. His course was uncomplicated with complete resolution of COVID-19 symptoms. He had no known drug allergies.

On presentation, he had a small pneumothorax with mild parenchymal changes on computed tomography (CT) but required intubation and mechanical ventilation 12 days after admission for declining respiratory status. Further worsening resulted in the institution of peripheral veno-venous extracorporeal membrane oxygenation (V-V ECMO) accompanied by paralysis and sedation 2 days after intubation. Subsequent radiographic evaluation showed extensive bilateral bronchiectasis, ground-glass opacities/consolidation, fibrotic changes, and honeycombing most prominent in the bilateral upper lobes (Figure 1A and B). At that time, he had been treated for *Enterococcus* bacteremia with a 14-day course of ampicillin. Given the temporal relationship between the patient’s drug exposure and the severity of his pulmonary function decline, confirmatory genetic testing was pursued and was significant for heterozygous allelic mutations predisposing to Bactrim allergy (human leukocyte antigen (HLA) B*07:02 and HLA C*07:02). The patient was eventually transferred to our institution for emergent evaluation and listing for lung transplantation.

**Figure 1:**
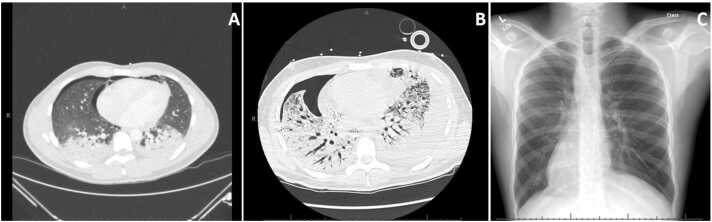
Chest CT (lung window). (A) Initial presentation after 1 month of Bactrim therapy; (B) on transfer after 2 months of Bactrim therapy; (C) post-transplant chest X-ray. CT, computed tomography.

On arrival, he was being treated with vancomycin for methicillin-resistant *Staphylococcus aureus* pneumonia. Despite optimized mechanical ventilation (6 ml/kg tidal volume, fraction of inspired oxygen (FiO_2_) 100%, peak end expiratory pressure (PEEP) 10) and extracorporeal life support (flow 4.38 liters, 3800 RPM, sweep 8, FdO2 100% via a single-site dual-lumen cannula in the right internal jugular vein), the patient's respiratory status worsened (hypoxemia with partial pressure of arterial oxygen (PaO_2_) 40-64 mm Hg) prompting conversion to central veno-arterial extracorporeal membrane oxygenation 7 days after his transfer. He underwent bilateral orthotopic lung transplantation with a standard criteria brain-death donor 1 day following his reconfiguration. The explant pathology showed diffuse mixed lymphoplasmacytic infiltration with patchy fibrosis, extensive septal thickening, and diffuse vascular thrombosis (Figure 2).

**Figure 2:**
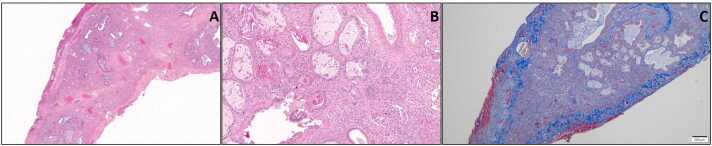
Histopathology from explanted lung. (A) H&E section (2× magnification) demonstrating interstitial fibrosis with interlobular septal and pleural thickening; (B) H&E section (10× magnification) demonstrating mixed lymphoplasmacytic infiltration in a background of fibrosis; (C) trichrome stain section (4× magnification) highlighting interstitial and subpleural fibrosis. H&E, hematoxylin and eosin stain.

His postoperative course was complicated by respiratory failure requiring a tracheostomy on postoperative day 10, left vocal cord paralysis managed with vocal fold injections, and heparin-induced thrombocytopenia managed with transition to bivalirudin for anticoagulation. He was liberated from V-V ECMO on postoperative day 6 and successfully discharged home 41 days following his transplantation with normal lung imaging (Figure 1C). Atovaquone is being used for post-transplant *Pneumocystis jirovecii* pneumonia prophylaxis given its lack of a sulfonyl group and demonstrated efficacy in prophylaxis.

Sulfonamides, such as Bactrim, are the second most common cause of DIILD, with male sex and pre-existing lung disease being common risk factors.^2^ The classic histopathology of Bactrim-induced lung injury is diffuse alveolar injury with delayed epithelization, resulting in interstitial fibrosis.^3^ Typical management of DIILD involves early supportive care and empiric use of glucocorticoids, often resulting in a good prognosis with resolution of radiographic findings.^2^, ^4^ Some rare cases, however, do progress to needing support and transplantation as reported here.

The patient had been diagnosed with COVID-19 infection a month before starting Bactrim. The association of COVID-19 infection and lung fibrosis is well described as having an insidious onset, with a dissimilar histopathology to diffuse alveolar injury with delayed epithelization. The interplay between fibrosis and external triggers beyond the virus is yet to be fully understood.^5^ The combination of contracting COVID-19 infection, coupled with the use of Bactrim in the setting of genetic susceptibility to Bactrim allergy, may have resulted in this fulminant pulmonary fibrosis ending in lung failure. The use of lung transplantation offers a viable treatment modality for patients who have exhausted conventional treatment options for lung failure secondary to DIILD.

DIILD is well described and classically managed expectantly. However, it can result in severe lung fibrosis, especially when priming factors are present, necessitating the use of advanced forms of respiratory support. It is crucial to be aggressive in the management of the worsening young patient because transplantation is a safe and viable therapeutic option.

## Author contributions

All authors contributed to the writing and editing of this manuscript.

## Patient consent

Authors confirm that appropriate patient consent to publish this case report was received.

## Disclosure statement

The authors declare that they have no known competing financial interests or personal relationships that could have appeared to influence the work reported in this paper.

Meeting presentation: 104th annual meeting of the America Association for Thoracic Surgery.
